# Blood Count-Derived Inflammatory Markers and Acute Complications of Ischemic Heart Disease in Elderly Women

**DOI:** 10.3390/jcm12041369

**Published:** 2023-02-08

**Authors:** Ewelina A. Dziedzic, Jakub S. Gąsior, Agnieszka Tuzimek, Wacław Kochman

**Affiliations:** 1 Cardiovascular Clinic, Centre of Postgraduate Medical Education, 01-813 Warsaw, Poland; 2 Department of Pediatric Cardiology and General Pediatrics, Medical University of Warsaw, 02-091 Warsaw, Poland

**Keywords:** acute coronary syndrome, monocyte-lymphocyte ratio, platelet-lymphocyte ratio, neutrophil-lymphocyte ratio, systemic inflammatory response index

## Abstract

**Simple Summary:**

Women are less likely to be correctly diagnosed with and subsequently treated for coronary artery disease (CAD). CAD is a result of atherosclerosis, a chronic disease of the arterial wall with a significant inflammatory component. Its complication, acute coronary syndrome (ACS), was described to be connected with inflammation in women more often than in men. Thus, a simple marker based on accessible laboratory tests, like total blood count, may be of great use. This paper describes an association between ACS occurrence and systemic inflammatory response index (SIRI), systemic inflammatory reaction index (SII), monocyte-lymphocyte ratio (MLR), platelet-lymphocyte ratio (PLR), and neutrophil-lymphocyte ratio (NLR), as well as it looks for the relationship between these and classic CAD risk factors. Our results suggest that with further well-designed research, the abovementioned markers may become a new CAD risk factor in elderly women.

**Abstract:**

Coronary artery disease (CAD) in women occurs later than in men. Underlying atherosclerosis, a chronic process of lipoprotein deposition in arterial walls with a prominent inflammatory component, is influenced by several risk factors. In women, commonly used inflammatory markers are generally found to be related to the occurrence of acute coronary syndrome (ACS), as well as the development of other diseases that influence CAD. New inflammatory markers derived from total blood count—systemic inflammatory response index (SII), systemic inflammatory reaction index (SIRI), monocyte-lymphocyte ratio (MLR), platelet-lymphocyte ratio (PLR), and neutrophil-lymphocyte ratio (NLR)—were analyzed in the group of 244 elderly, postmenopausal women with the diagnosis of ACS or stable CAD. SII, SIRI, MLR, and NLR were significantly higher in women with ACS compared to those with stable CAD (*p* < 0.05 for all)—the highest values were observed in women with NSTEMI. MLR from new inflammatory markers, HDL, and history of MI turned out to be significant factors associated with ACS. These results suggest that MLR as representative of blood count-derived inflammatory markers may be considered as additional CVD risk factors in women with suspected ACS.

## 1. Introduction

World Health Organization data indicate that cardiovascular disease (CVD) is the cause of mortality in 55% of European women [1]. In this group, coronary artery disease (CAD) occurs on average 10 years later compared to men [2]. The delay of a few years in the occurrence of major adverse cardiovascular events (MACE) is suggested to be due to the cardioprotective influence of estrogen, in particular its anti-inflammatory qualities [3]. Endogenous estrogen deficiency causes a seven-fold increase in CVD risk [4]. Moreover, the 17β-estradiol concentration is inversely proportional to the number of MACE in the postmenopausal period [5]. In addition to the discrepancy in the age of the diagnosed sex groups, differences were also observed in CVD risk factors and inflammatory markers [6]. Comparative analysis between sex groups revealed that 37 of 71 serum CVD biomarkers were higher in women than in men, indicating the important role of systemic inflammation and its complications in this group [7]. A higher level of C-reactive protein (CRP) [8], a systemic inflammation marker, and a decreased inflammatory response to stimuli [9] was observed in women independently of the present CVD risk factors. In a group of healthy middle-aged and elderly female responders, the baseline concentration of CRP was found to be an independent predictor of future cardiovascular events [10]. Increased levels of inflammatory biomarkers correlate not only with a higher risk of atherosclerosis and its complications, but also with an increased chance of developing other CVD risk factors in the future [8,11]. Elevated expression of inflammatory cytokines (CRP, Il-6, TNF-α) in a period of observation of several years increased the risk of insulin resistance [12], diabetes, and metabolic syndrome [11]. The MESA study described a positive correlation between CRP concentrations and the risk of developing hypertension [8]. Analysis of recent data on the influence of classic CVD risk factors revealed a more pronounced effect on the occurrence of ACS in women compared to men with an odds ratio of 1.6, 1.5, and 1.3 for diabetes, hypertension, and smoking, respectively [8]. Furthermore, some nontraditional CVD risk factors specific to women were found, e.g., unfavorable pregnancy results, autoimmune diseases, breast cancer treatment, and depression [13].

Due to a smaller representation of female patients in the research [14], there is a possibility of an underestimation of the CVD risk and frequency of CAD in this group [15]. Late diagnosis and less aggressive CVD treatment result in worse effects of percutaneous interventions (PCI) and frequent need to repeat this procedure [16]. Subsequently, early and late in hospital mortalities were also higher [17]. Due to this, early diagnosis and treatment of ACS appear to be more challenging in women compared to men [18]. 

Atherosclerosis, the underlying cause of CVD, is a chronic disease with a prominent inflammatory component [19]. Taking this into account, the markers of subclinical systemic inflammation are of particular interest, especially those that incorporate different subtypes of immune cells. In cardiologic patients, markers employing two cell lines, such as monocyte-lymphocyte ratio (MLR), platelet-lymphocyte ratio (PLR), and neutrophil-lymphocyte ratio (NLR) were shown to be good evaluators of CVD and mortality risk [20,21,22]. Recently, markers employing three cell lines, such as systemic inflammatory response index (SIRI) and systemic inflammatory index (SII), have been investigated. SIRI was found to be associated with the risk of ACS in patients with chest pain [23], as well as with MACE in patients with a history of PCI [24] or the occurrence of supraventricular tachycardia in patients with stroke [25]. Similarly, SII is positively associated with the severity of CAD [23,26], the risk of massive pulmonary embolism [27], and the probability of MACE in patients with heart failure [28], after cardio surgery [29,30] or PCI [24]. In postmenopausal patients, SII was analyzed in the context of mineral bone density and the risk of osteoporosis [31,32] or bone fracture [33]. Furthermore, in this group of patients, correlations between NLR and arterial stiffness were found [34], as well as between PLR and angioneurotic symptoms [35]. 

Due to different inflammatory responses, the diversity of symptoms and clinical course of CVD in both sexes points to the need to find new risk factors for ACS in women. This research aims to analyze the correlation between new biomarkers, SII, SIRI, MLR, NLR, PLR, and ACS in elderly, postmenopausal women.

## 2. Materials and Methods

### 2.1. Population and Clinical Data, Exclusion Criteria 

The database of Polish patients referred for CAD evaluation with catheter angiography between 2013 and 2017 was filtered to distinguish cases that met the inclusion criteria—women over 50 years of age. All participants in the database agreed in writing to use their data in the study. Exclusion criteria were elevated inflammatory markers (white blood cell count (WBC) > 10,000 cells/μL, elevated erythrocyte sedimentation rate, CRP > 5 mg/L), active neoplasia, viral or bacterial infections, paraneoplastic syndromes. 

### 2.2. Measurements 

Body mass index (BMI, kg/m^2^) was calculated using weight and standing height, smoking status and laboratory data was obtained by standard clinical-chemical assays in the hospital laboratory from fasting blood samples obtained by antecubital venipuncture during the admission process. Diabetes, hyperlipidemia, and hypertension were diagnosed using criteria from the respective recommendations [36,37,38] and are summarized in Table 1.

All markers based on WBC count subtypes were calculated using the same blood sample measurement with the formulas as follows: NLR, PLR, and MLR as ratios of neutrophils, platelets, and monocytes to lymphocytes, respectively. SII was determined as neutrophils × platelets/leukocytes, and SIRI as neutrophils × monocytes/lymphocytes.

ACS was diagnosed if an increase in myocardial necrosis markers (especially troponin) was accompanied by at least one of the following: myocardial ischemia symptoms, recent signs of ischemia or pathological Q waves on the ECG, a new loss of viable myocardium in imaging studies, or a new segmental disturbance in heart wall movement, a coronary artery thrombus on angiography [39].

### 2.3. Statistical Analysis

The data distribution was evaluated with the Kolmogorov-Smirnov test. The chi-square statistic was used to identify associations between dichotomous and categorical data. Continuous variables between the two groups were compared using the Mann-Whitney test or the t test. The Kruskal-Wallis one-way analysis-of-variance-by-ranks test (H) was used to determine significant differences in analyzed parameters between different patient subgroups. Binary multivariable logistic regression was employed to identify factors associated with the outcome variable. Model fitness was checked by using the Hosmer–Lemeshow goodness of fit test. To express the performance of the logistic regression models, the area under the curve (AUC) statistic was used. The Spearman rank correlation coefficient was used to analyze the relationship between selected biomarkers. The receiver operating characteristic (ROC) curve analysis was used to identify the optimal cut-off values of markers according to the Youden index (maximum = sensitivity + specificity − 1). A *p*-value < 0.05 was considered statistically significant. The software used for the analysis and figures was Statistica 13 (StatSoft Inc., Tulsa, OK, USA) GraphPadPrism 5 (GraphPad Software Inc., San Diego, CA, USA, 2005), and PQStat 1.8.4 (PQStat Software, Poznan, Poland), respectively.

## 3. Results

### 3.1. Population Characteristics

The results of 244 elderly, postmenopausal women were included in the final statistical analysis. The mean age of the study population was 72.0 years (SD: 9.1). The median BMI value was 28.3 kg/m^2^ (IQR: 20.7–38.1). Sixty-six (27.0%) participants had a normal body weight, 76 (31.1%) were overweight, and 88 (36.1%) were classified as obese. A history of type 2 diabetes mellitus (t2DM) or diagnosis during the current hospitalization was found in 83 (34.0%) patients and pre-diabetes in 9 (3.7%) patients. On basis of the lipid profile (total cholesterol—TC, LDL and HDL cholesterol, triglycerides—TG), hyperlipidaemia was assessed in 229 patients and was diagnosed in over half of them despite statin treatment, i.e., in 136 (55.7%). Hypertension was present in 212 (86.9%) patients. A history of myocardial infarction (MI) was noticed in 83 (34.0%) patients. Active smoking during the study was declared by 43 (17.6%) patients. Median (IQR) for the following parameters was: TC: 178.9 mg/dL (114.3–279.8); HDL: 52.0 mg/dL (34.7–81.3); LDL: 97.6 mg/dL (49.4–190.9); TG: 116.8 mg/dL (54.7–230.7); PLT: 225.5 mcL (161.0–353.0); neutrophils: 4.7 thousand cells/µL (2.6–9.3); monocytes: 0.7 thousand cells/µL (0.4–1.2); lymphocytes: 1.9 thousand cells/µL (0.9–3.2); NLR: 2.5 (1.1–6.9); MLR: 0.3 (0.2–0.9); PLR: 119.9 (67.1–222.5), SII: 547.3 (223.7–1594.7) and SIRI: 1.6 (0.6–7.4). 

### 3.2. Difference in Selected Parameters and Analysed Biomarkers between Patients with Stable CAD and Patients with ACS

**Figure 1:**
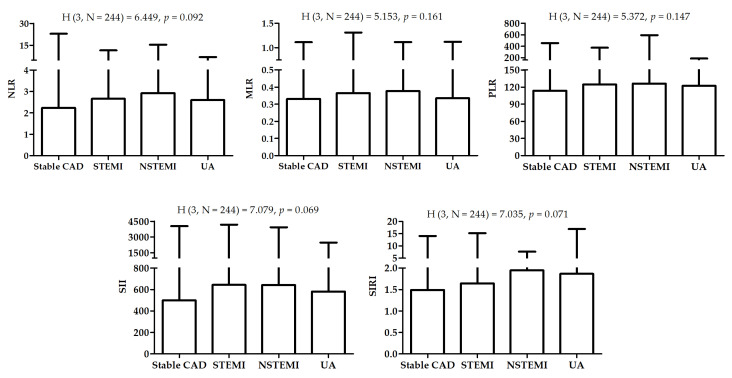
Differences in biomarkers between patients with different diagnosis. H—the Kruskal-Wallis one-way analysis-of-variance-by-ranks test.

Acute coronary syndrome (ACS) as the cause of hospitalization was diagnosed in 109 (44.7%) patients, while stable CAD was the cause in 135 (55.3%) patients. Significant differences were observed between patients with ACS and stable CAD in HDL, NLR, MLR, SII, and SIRI. There was a significant disproportion of patients with a history of MI between the two groups (Table 2). Figure 1 presented differences in biomarkers between patients with different diagnoses.

### 3.3. Factors Associated with ACS Diagnosis

The results of the logistic regression analysis of factors associated with ACS diagnosis (based on results presented in Table 2) are presented in Table 3. A history of MI and HDL was associated with ACS diagnosis. The Hosmer-Lemeshow goodness-of-fit test produced a test statistic of 13.308 (with a *p*-value of 0.102). The AUC of the regression model was 0.751.

Correlation coefficients between biomarkers are presented in Table 4 (*p* for all < 0.001). Due to the observed collinearity, to generate a final model, elimination by backward stepwise multivariable logistic regression was additionally performed (Table 5). History of MI, HDL, but also MLR were associated with ACS. The Hosmer-Lemeshow goodness-of-fit test produced a test statistic of 10.046 (with a *p*-value of 0.262). The AUC of the regression model was 0.744.

**Figure 2:**
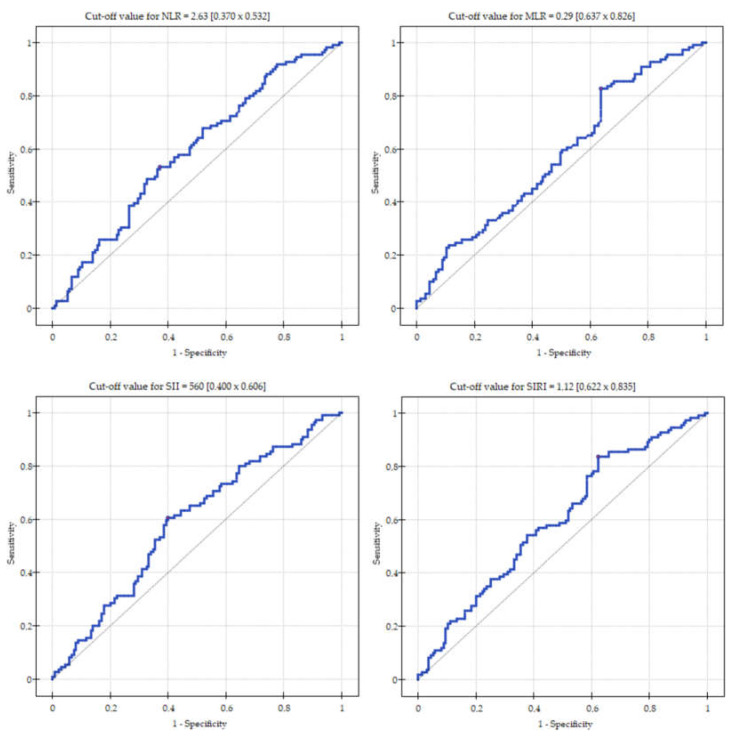
Receiver operating characteristics curve analysis for NLR, MLR, SII and SIRI.

Cut-off values, corresponding sensitivity, and specificity for the NLR, MLR, SII and SIRI are presented in Figure 2. The area under the curve (AUC) for the biomarkers was as follows: 0.589 (95%CI: 0.518–0.661, *p* = 0.017) for NLR; 0.574 (95%CI: 0.503–0.646, *p* = 0.046) for MLR; 0.590 (95%CI: 0.519–0.662, *p* = 0.016) for SII and 0.597 (95%CI: 0.526–0.669, *p* = 0.009) for SIRI.

## 4. Discussion

This research describes the association between systemic inflammatory markers based on WBC count and the occurrence of ACS in elderly, postmenopausal women. The majority of markers, NLR, MLR, SII, and SIRI (but not PLR), were significantly higher in women with ACS compared to those with stable CAD. Patients with subtypes of ACS (STEMI, NSTEMI, UA) had similar values, but women with NSTEMI presented nominally the highest values of the markers investigated. Despite higher values of these markers in women diagnosed with ACS, they seem to be insignificant when compared to known risk factors of ACS. However, all were correlated with each other. Backward stepwise regression analysis allowed us to identify MLR from new inflammatory markers, HDL, and history of MI as significant factors associated with ACS. 

Previously, we found an association between higher SII and SIRI with diagnosed ACS in a cohort of nearly 700 patients with chest pain [23]. Furthermore, analysis of patients with a history of myocardial infarction revealed higher values of NLR in patients with consecutive ACS compared to those with stable CAD and a history of myocardial infarction [40]. However, all groups had similar platelet activity parameters (MPV and P-LCR) [41]. These articles are parts of a project on the association of blood cell count with acute complications of atherosclerosis. 

CVD is responsible for the majority of lost life years in women, and in middle-income countries, it is responsible for 43% of mortality in this group. ECS data report 1.6 million new cases in women every year [1]. This epidemic is caused by atherosclerosis, a chronic inflammatory disease responsible for an accumulation of lipoproteins in the wall of medium and large arteries [42,43]. This complicated process comprising the influence of both CVD risk factors and the immune system, including leukocyte migration, leads to plaque formation [44]. Previously, WBC and their subtypes were shown to participate in localized and systemic immune responses. Its count correlates with a higher risk of CVD [45,46,47] and is an independent predictor of mortality after ACS [48]. 

A reaction to endothelial injury includes the recruitment of monocytes from circulating blood to the intima media of the arteries [49]. Macrophages, the final form of monocytes in the vessel wall, are the most prominent fraction in atherosclerotic plaques [44]; and their number depends on the hematopoietic activity of the bone marrow, as well as local proliferation and concentrations of integrin and chemokine receptors involved in this process [50]. Their distinct phenotypes have different functions in the atherosclerotic process [51]. The M1 type produces pro-inflammatory factors, including NO and reactive oxygen species, and promotes monocyte recruitment and the progression of atherosclerosis [52]. The M4 type has anti-inflammatory qualities, which inhibit atherogenesis [53]. Both of these subtypes may be responsible for subsequent ACS despite optimal pharmacotherapy in patients with myocardial infarction in their history [54]. Furthermore, the number of monocytes is an independent risk factor for CVD mortality in short-term [55] and long-term observation [56]. Neutrophils have an influence on arterial wall inflammation [57], as their number correlates with the size of atherosclerotic plaque and the risk of MACE in the future [58]. They influence monocyte migration [59] through chemotactic proteins and extracellular traps, which stimulate macrophages to produce IL-1β [60], and regulate the rupture potential of plaque [61]. By affecting thrombocyte and endothelial function, they shape the thrombotic and injury potential of microcirculation [62]. An important role for inflammation in plaque destabilization and breakage was confirmed by the several times higher risk of MACE observed after respiratory tract infections [63] due to hyperacute monocyte recruitment and increased neutrophil infiltration in arterial walls [64]. 

Lymphocytes are another group of cells that modifies the progress of atherosclerosis [65]. T CD4+ cells, common in plaques, start an immune reaction signaled by autoantigens on apolipoprotein B100 in low-density lipoproteins [66]. Type 1 T-helper cells promote atherogenesis, T-regulating cells have a preventive function, and type 17 T-helper cells stimulate plaque fibrosis and stabilization [65]. In some circumstances, T-regulating cells can be proatherogenic—previous research described the correlation between lymphopenia and MACE [67], poor prognosis after myocardial infarction [68], and in patients with heart failure [69]. 

Platelets play a well-documented role in atherogenesis, as they connect inflammation with thrombosis [70]. They are activated by endothelial injury to produce chemokines that initiate and maintain inflammation [71]. Furthermore, platelet activity markers correlate with thrombotic potential [72]. 

Considering the influence of inflammation and various immune cell mechanisms involved in the initiation, progression, destabilization, and rupture of atherosclerotic plaque, markers comprising a few immune pathways are of particular interest, as they appear to represent the balance between inflammation and immune response. Here, we describe the correlation between markers, including two (NLR, MLR) and three (SII, SIRI) cell lines with ACS in elderly women. The results of recent studies of a mixed population (women and men) indicate the prognostic value of the analyzed markers to predict early (12-month) restenosis after carotid endarterectomy [73], obstruction after revascularization of the lower extremities [74] and poor clinical condition in patients after elective coronary angioplasty [75]. Furthermore, the positive additive interaction of serum uric acid concentration and NLR value was shown to correlate with reinfarction and death from cardiovascular causes [76]. These inflammatory markers were also analyzed in the context of osteoarticular diseases.

In postmenopausal women, higher values of NLR and SII correlate with mineral bone density and osteoporosis [31,32]. MLR, PLR, and SIRI were described as risk factors for fractures [33]. Furthermore, in this group, higher NLR was correlated with arterial stiffness, an indicator of subclinical atherosclerosis [34]. Taking into account the proposed association between CVD and bone metabolism [77], as well as the recent gene analysis pointing to an immune and inflammatory response as a shared factor in the pathophysiology of both atherosclerosis and osteoporosis [78], the above-mentioned research suggests a correlation with bone mass density and osteoporosis appear to corroborate our results. The lack of connection between PLR and ACS may result from including only platelet and lymphocyte counts, contrary to other markers that comprise monocytes and neutrophils with the functions described above. 

The results obtained in this research indicate that the new, affordable, and accessible systemic inflammatory markers may be an important risk factor for ACS in elderly women. A large study TACTICS-TIMI in patients with UA or NSTEMI described that elevated troponin levels were more common in men compared to women, but elevated CRP was following a reverse trend [79]. Due to this, we suggest considering blood count-derived inflammatory markers as additional CVD risk factors in women with suspected ACS. Furthermore, the data presented confirm the role of subclinical inflammation in acute CAD complications in female patients, who, compared to men, are less likely to receive statin treatment and receive a smaller dose of it [80].

The limitations of this research include the cross-sectional, observatory character, which forbids causational investigation between the data. Furthermore, the number of included patients is limited. The inclusion criteria included women over 50 years of age, however, the hormonal function of the ovaries was not determined, and the intake of hormone replacement therapy was not verified. Patients with elevated standard inflammatory markers (WBC, CRP) were excluded from the study; the ferritin or inflammatory cytokine (TNF-alpha, IL-6) concentrations were not measured, however. Other factors, such as the dose of ingested lipid lowering drugs, diabetes control, smoking, and comorbidities were not taken into account. 

The relevance of systemic markers of subclinical inflammatory processes in CVD is under investigation and needs further well-designed research.

## 5. Conclusions

Higher inflammatory markers (NLR, MLR, SII, SIRI) were found in elderly, postmenopausal women with ACS compared to those with stable CAD. MLR may be considered as additional CVD risk factors in women with suspected ACS. The role of biomarkers based on WBC count in CVD requires further well-designed research.

## Figures and Tables

**Table 1:** Criteria for diagnosing diabetes, hyperlipidemia, dyslipidemia, and hypertension in patients.

Diagnosis	Diagnostic Criteria
Diabetes [36]	Fasting plasma glucose concentration ≥ 126 mg/dL twice or random plasma glucose measurement > 200 mg/dL
Hyperlipidemia [37]	TC > 200 mg/dL and/or TG > 150 mg/dL.
Dyslipidemia [37]	LDL > 70 mg/dL, HDL < 50 mg/dL
Hypertension [38]	SBP > 140 mmHg and/or DBP > 90 mmHg

TC—total cholesterol, TG—triglycerides, LDL—low-density lipoproteins, HDL—high-density lipoproteins, SBP—systolic blood pressure, DBP—diastolic blood pressure.

**Table 2:** Differences in selected parameters between patients with ACS and stable CAD.

Variable	ACS	Stable CAD	*p*-Value
Age [years]	73.1 ± 9.9	71.1 ± 8.3	0.086
BMI [kg/m^2^]	28.2 (19.5–38.3)	28.4 (21.2–38.1)	0.927
t2DM (no/yes/pre-diabetes)	65/40/4	87/43/5	0.726
Hyperlipidaemia (no/yes) *	36/67	57/69	0.114
HDL [mg/dL] *	48.1 (30.4–71.0)	57.2 (37.6–92.1)	<0.001
LDL [mg/dL] *	104.7 (47.9–196.7)	90.3 (50.0–165.6)	0.054
TG [mg/dL] *	109.4 (53.5–225.5)	123.4 (56.9–257.2)	0.076
TC [mg/dL] *	178.9 (114.1–283.8)	179.7 (119.3–256.7)	0.701
Hypertension (no/yes)	12/97	20/115	0.381
History of MI (no/yes)	53/56	108/27	<0.001
Smoking (no/yes)	80/26	102/17	0.051
Platelet (PLT) [mcL]	229.0 (164.0–361.0)	220.0 (158.0–353.0)	0.277
Neutrophils [thousand cells/µL]	5.0 (2.8–9.5)	4.6 (2.3–8.0)	0.101
Monocytes [thousand cells/µL]	0.7 (0.4–1.3)	0.7 (0.4–1.1)	0.111
Lymphocytes [thousand cells/µL]	1.8 (0.9–3.2)	2.0 (0.9–3.5)	0.153
NLR	2.7 (1.3–6.9)	2.2 (0.9–7.3)	0.017
MLR	0.4 (0.2–1.0)	0.3 (0.2–0.7)	0.046
PLR	124.6 (70.9–236.8)	113.6 (63.0–218.0)	0.096
SII	610.0 (268.0–1626.9)	499.3 (179.7–1594.7)	0.016
SIRI	1.9 (0.7–7.7)	1.5 (0.6–5.4)	0.009

*—assessed in 229 patients.

**Table 3:** Multivariable logistic regression analysis of factors associated with ACS diagnosis.

Variables	Category	β	Wald Stat. (95% CI)	Odds Ratio (95% CI)	*p*-Value
History of MI	Yes	1.318	17.39 (0.69–1.94)	3.73 (2.01–6.94)	<0.001
HDL [mg/dL]	-	−0.047	16.59 (−0.07–−0.02)	0.95 (0.93–0.98)	<0.001
NLR	-	−0.178	0.91 (−0.54–0.19)	0.84 (0.58–1.21)	0.339
MLR	-	2.228	2.31 (−0.65–5.10)	9.28 (0.52–164.36)	0.129
SII	-	0.001	0.78 (−0.001–0.002)	1.00 (0.99–1.00)	0.377
SIRI	-	−0.036	0.06 (−0.34–0.26)	0.96 (0.72–1.30)	0.812

**Table 4:** Correlation coefficient between analyzed biomarkers.

	NLR	MLR	SII	SIRI
NLR	X			
MLR	0.730	X		
SII	0.894	0.658	X	
SIRI	0.868	0.869	0.816	X

**Table 5:** Final model of backward stepwise multivariable logistic regression analysis of factors associated with ACS diagnosis.

Variables	Category	β	Wald Stat. (95% CI)	Odds Ratio (95% CI)	*p*-Value
History of MI	Yes	1.313	17.46 (0.69–1.93)	3.72 (2.01–6.89)	<0.001
HDL [mg/dL]	-	−0.047	17.18 (−0.07–−0.02)	0.95 (0.93–0.98)	<0.001
MLR	-	1.728	5.73 (0.31–3.14)	5.63 (1.37–23.16)	0.017

## Data Availability

Data can be provided by the corresponding author upon reasonable request.
